# Cases

**Published:** 1891-11

**Authors:** Thomas B. Rogers


### Laryngismus Stridulus.

By Thomas B. Rogers, D. V. S.

The Fleming operation for "roaring" does not seem to make much headway; almost all operators who have tried it report unfavorably on it. The operation of Mœller is difficult; it is one thing to talk about dissecting a mucous membrane from the arytenoid cartilage, quite another to do it successfully even on the cadaver. In most animals past their prime it is in part adherent and it cannot well be removed from Santorini's cartilage. It is true that the procedure suggested by Fleming, is antiquated in regard to the after care and antiseptics and this will necessarily be unfavorable with regard to the exuberance of granulations during healing.

Shall we then allow the condition of the larynx induced by left recurrent paralysis to be a *noli me tangere* of surgery? Is there no other way to restore these often valuable animals to their pristine usefulness? The writer believes that a remedy will be found in nerve grafting.

There is no operative difficulty in the way to prevent the distal end of the left recurrent laryngeal nerve being grafted on the right recurrent in the middle of the neck, and if muscle cells still remain intact in the atrophied crico-arytenoidei postici muscles there is no reason why the muscle should not in time become regenerated when supplied with healthy nerve stimulus.

Most surgeons of experience have seen the marked atrophy of the anterior crural region following peptone poisoning (azoturia) when the crural triceps muscle, the fascia lata and the anterior gracilis of the thigh disappear almost entirely through pressure on their nerves of supply, and gradually recover their tone and substance when the pressure on the nerves is removed and the current resumed. The same thing occurs in the aphonia of diphtheria in man.

I quote a case in point to show that there is no operative difficulty in the way: "A lacerated wound of the left arm had torn the median nerve. Deprès found it impossible to bring the central end of the torn nerve down so as to suture it to its corresponding fragment. *He therefore exposed the ulnar nerve and, having separated its fibres by tearing them apart with a pair of dressing forceps, and into the interspaces inserted the fibres of the peripheral end of the median nerve. The procedure was crowned with success and the patient recovered a useful hand.** 

I believe that experiment on this line will bear fruit; and make the idea public in the hope that many surgeons will have many opportunities, while but few come to the individual. It cannot make a bad roarer much worse, and subjects the patient to no risk of life or remaining usefulness.

* Gazette Hebdom. de Méd. et de Chir., No. 5, 1876.

### FOREIGN BODIES IN THE ŒSOPHAGUS OF BOVINES.

By Same.

On two occasions when attempting the removal of foreign bodies from the superior portion of the œsophagus of the cow the animal expired suddenly without any warning or difficulty of breathing. The obstruction could just be reached with the fingers and the left hand was in the œsophagus while the right pressed upward the foreign body. Now, when I fail to readily remove the foreign body through the mouth, it is my practice to cut down on the œsophagus, raise it from its bed and after introducing a little oil orally, to press the offending substance upward. The operation is easy, devoid of risk, and does away with the bruising of the parts, and the arm of the surgeon which usually follow prolonged efforts at oral extraction.

---

### DIVISION OF THE CUNEAN BRANCH OF THE FLEXOR OF THE METATARSUS FOR SPAVIN.

By Same.

On two occasions this operation has been followed in my practice by enormous increase of the bony growth, and no amelioration of the lameness. In cases where, after firing and rest, there was remaining lameness, the operation has been entirely successful. I believe the place of this operation is as an adjuvant to the classical treatment, but as my experience is yet limited should be glad to hear the experience of others.

